# Absence of zoonotic *Bartonella *species in questing ticks: First detection of *Bartonella clarridgeiae *and *Rickettsia felis *in cat fleas in the Netherlands

**DOI:** 10.1186/1756-3305-4-61

**Published:** 2011-04-18

**Authors:** Ellen Tijsse-Klasen, Manoj Fonville, Fedor Gassner, Ard M Nijhof, Emil KE Hovius, Frans Jongejan, Willem Takken, Johan R Reimerink, Paul AM Overgaauw, Hein Sprong

**Affiliations:** 1 Laboratory for Zoonoses and Environmental Microbiology, National Institute for Public Health and Environment (RIVM), Bilthoven, The Netherlands; 2 Laboratory for Infectious Diseases and Screening, National Institute for Public Health and Environment (RIVM), Bilthoven, The Netherlands; 3 Laboratory of Entomology, Wageningen University, Wageningen, The Netherlands; 4 Companion Animal Hospital 't Heike, Veldhoven, The Netherlands; 5 Institute for Risk Assessment Sciences, Division of Veterinary Public Health, Utrecht University, Utrecht, The Netherlands; 6 Utrecht Centre for Tick-borne Diseases (UCTD), Department of Infectious Diseases and Immunology, Faculty of Veterinary Medicine, Utrecht University, Utrecht, The Netherlands; 7 Institute for Parasitology and Tropical Veterinary Medicine, Freie Universität Berlin, Berlin, Germany

## Abstract

**Background:**

Awareness for flea- and tick-borne infections has grown in recent years and the range of microorganisms associated with these ectoparasites is rising. *Bartonella henselae*, the causative agent of Cat Scratch Disease, and other *Bartonella *species have been reported in fleas and ticks. The role of *Ixodes ricinus *ticks in the natural cycle of *Bartonella *spp. and the transmission of these bacteria to humans is unclear. *Rickettsia *spp. have also been reported from as well ticks as also from fleas. However, to date no flea-borne *Rickettsia *spp. were reported from the Netherlands. Here, the presence of *Bartonellaceae *and *Rickettsiae *in ectoparasites was investigated using molecular detection and identification on part of the gltA- and 16S rRNA-genes.

**Results:**

The zoonotic *Bartonella clarridgeiae *and *Rickettsia felis *were detected for the first time in Dutch cat fleas. *B. henselae *was found in cat fleas and *B. schoenbuchensis *in ticks and keds feeding on deer. Two *Bartonella *species, previously identified in rodents, were found in wild mice and their fleas. However, none of these microorganisms were found in 1719 questing *Ixodes ricinus *ticks. Notably, the gltA gene amplified from DNA lysates of approximately 10% of the questing nymph and adult ticks was similar to that of an uncultured *Bartonella-*related species found in other hard tick species. The gltA gene of this *Bartonella-*related species was also detected in questing larvae for which a 16S rRNA gene PCR also tested positive for "*Candidatus *Midichloria mitochondrii". The gltA-gene of the *Bartonella-*related species found in *I. ricinus *may therefore be from this endosymbiont.

**Conclusions:**

We conclude that the risk of acquiring Cat Scratch Disease or a related bartonellosis from questing ticks in the Netherlands is negligible. On the other hand fleas and deer keds are probable vectors for associated *Bartonella *species between animals and might also transmit *Bartonella *spp. to humans.

## Background

*Bartonella *species are facultative intracellular Gram-negative bacteria which can infect humans and a wide range of animal species. Cat-scratch disease (CSD) is probably the most common *Bartonella *infection in the northern hemisphere [1,2]. The hallmark of CSD is enlargement and tenderness of lymph nodes draining the site of inoculation of the microorganism [3]. Regional lymphadenopathy usually develops 2 to 3 weeks after exposure and normally resolves spontaneously after several months [4]. Thirty percent of the patients report low-grade fever and a skin or mucous membrane lesion may be observed at the site of inoculation for 25% to >90% of patients [3,5]. Extranodal clinical manifestations, such as encephalopathy, neuroretinitis, arthritis, and lytic bone lesions, occur in approximately 10% of patients [3,5-8]. Furthermore, *Bartonella *spp. are an important cause for blood-culture negative endocarditis [9-11]. Disease symptoms depend on the immune status of the host; in immuno-compromised hosts, the bacteria are often present in blood and involved in angioproliferative disorders such as bacillary angiomatosis and peliosis hepatis [12]. The domestic cat is the major reservoir of *Bartonella henselae*, with a confirmed link to disease in humans [13,14]. Infected cats are usually asymptomatic and develop relapsing bacteraemia for long periods [15]. In a Dutch study, half of the cats were serological positive, and 22% were found to have a *B. henselae *bacteremia [16]. Bites or scratches from infected cats are associated with development of CSD. Cat fleas, *Ctenocephalides felis*, are involved in transmission between cats and may be also able to transmit *B. henselae *to humans: Approximately 30% of patients with CSD do not recall traumatic cat contact [17-19]. Solid evidence to support transmission via cat fleas is lacking. Other *Bartonella *species, including *Bartonella clarridgeiae *and *Bartonella grahamii*, have also been linked to human disease. However, there are only few reports of disease cases linked to these species [20-23].

The number of cases with tick-borne diseases in the Netherlands is on the rise [24]: This is illustrated by the fourfold increase in reported cases of erythema migrans since 1994, up to 22,000 patients in 2009. The most straightforward explanation is the reported increase in the incidence of tick bites [24]. The same tick species transmitting the etiologic agents of Lyme disease may also serve as vector of the causative agent of CSD and maybe also other *Bartonella *species [25-27]. Several PCR-based studies have demonstrated *B. henselae *DNA in various Ixodid tick species [26,28-32]. A recent study demonstrated that *B. henselae *can be transmitted across the developmental stages of *Ixodes ricinus *[33]. Altogether, these studies imply that CSD can be acquired from ticks, but studies contradicting this conclusion have also been published [34]. One of the aims of this study is to investigate whether *Bartonellaceae*, particularly *B. henselae*, are present in *I. ricinus *ticks in the Netherlands and form a risk to public health.

Another class of pathogens that can be transmitted by both Ixodid ticks and cat fleas is *Rickettsiae*. They are fastidious, mostly obligate intracellular alpha-proteobacteria. Hard ticks (Ixodidae) have been identified as vectors of the spotted fever syndrome in humans, which is caused by at least 15 different *Rickettsia *species [35]. Two infamous members of this group are *Rickettsia rickettsii*, the causative agent of Rocky Mountain spotted fever, and *Rickettsia conorii*, the causative agent of Mediterranean spotted fever [36]. *Rickettsia helvetica is *the most prevalent rickettsial species found in *I. ricinus *ticks in the Netherlands [37,38]. To date, the pathogenic potential of *R. helvetica *is unclear but infection with *R. helvetica *has been suspected in acute perimyocarditis, unexplained febrile illness, sarcoidosis and recently also meningitis [39-47]. Laboratory diagnosis of rickettsioses is predominantly based on serology. Currently, micro-immunofluorescence is considered as a reference serological assay, but most commercially available tests offer a very limited set of antigens, mostly *R. rickettsii *and/or *R. conorii*, and serological cross-reaction with other rickettsial pathogens are common. Notably, some patients suspected to have (suffered from) a (mild) rickettsiosis do not recall a tick bite. In these cases, other potential sources of infection, for example cat fleas, might be involved. Cat fleas may maintain and transmit *Rickettsia felis *which is the causative agent of flea-borne spotted fever, also called cat flea typhus or summer flu [48]. Clinical signs are similar to those of murine typhus and other febrile illnesses [49]. Patients usually have fever, fatigue, headache, myalgia, rash and elevation of liver enzymes, although these clinical manifestations do not occur in all patients. They can also present abdominal pain, pleuric chest pain, diarrhea, nausea, vomiting, conjunctivitis, and neurological symptoms [50-54]. *R. felis *appears to have a global distribution [48], and it is not unlikely that *R. felis *is also present in the Netherlands. Nevertheless, evidence for the presence of *R. felis *in Dutch cat fleas is lacking and no autochthonous clinical cases of flea-borne rickettsisos have been reported. In this study, we investigated whether *R. felis *is present in Dutch cat fleas.

## Methods

### Collection of ticks, fleas, deer keds and wildlife samples

Nymphs and adult ticks were collected for recent studies between 2006 and 2010 by flagging vegetation at 16 different locations in The Netherland (Table 1). Additionally, questing ticks from all stages were collected from vegetation in Vrouwenpolder in October 2010.

**Table 1:** Geographic distribution of questing *I. ricinus *ticks (nymphs and adults)

Location	(Previous study)	Ticks (n)	*Bartonella-*related *spp. (EF662054)*
Apeldoorn	Gassner *et al*., 2010 [74]	40	4
Bergherbos	Tijsse-Klasen *et al*., 2010 [38]	17	0
Duin en Kruidberg	Sprong *et al*., 2009 [37]	157	36
Ede	Gassner *et al*., 2010 [74]	271	14
Eijsden	Gassner *et al*., 2010 [74]	49	2
Gieten	Gassner *et al*., 2010 [74]	22	1
Haaksbergen	Gassner *et al*., 2010 [74]	15	0
Heumensoord	Tijsse-Klasen *et al*., 2010 [38]	67	5
Hoog Baarlo	Gassner *et al*., 2010[74]	221	12
Hullenberg	Tijsse-Klasen *et al*., 2010 [38]	60	6
Kwade Hoek	Gassner *et al*., 2010 [74]	122	15
Leusderheide	Tijsse-Klasen *et al*., 2010 [38]	140	12
Twiske	Gassner *et al*., 2010 [74]	167	25
Veldhoven	Gassner *et al*., 2010 [74]	16	1
Wassenaar	Gassner *et al*., 2010 [74]	67	3

Vrouwenpolder	This study	96	41

**Total**		**1527**	**177**

Tick lysates were tested for the presence of *Bartonella *DNA using the gltA-primers. Samples were analysed by gel electrophoresis. PCR products of 380 bp were sequenced. Only *Bartonella-related *DNA sequences <99% similar to EF662054 were detected.

Ticks from cats were collected by veterinarians between 2006 and 2009 as described [55]. More than 200 veterinarian clinics expressed interest in the study and were supplied with information packages containing posters, brochures and collection tubes. Participating clinics were asked to record host species, residence area of the pet owner, date of collection and whether the pets had travelled outside the Netherlands recently.

Deer keds (*Lipoptena cervi*) and attached ticks were collected from red deer by hunters.

All ticks used for this study were identified as *Ixodes ricinus *according to current keys, and life stage and sex of the ticks were recorded.

Tissue samples (ears) were collected from wood mice caught in Duin and Kruidberg area [37].

Fleas from bank voles (*Myodes glareolus*) and wood mouse (*Apodemus sylvaticus*) were derived from a previous study [37].

Fleas from 109 cats and 44 dogs were collected by 15 veterinary clinics and volunteers throughout the Netherlands between October 2009 and October 2010. Fleas were identified according to current taxonomic keys and pools of fleas were formed per host animal and per flea species.

Ticks, fleas and deer keds were immersed in 70% ethanol immediately after collection and stored at -20°C until DNA extraction.

### DNA extraction

DNA from vegetation ticks, dear keds and fleas collected from cats and dogs was extracted by alkaline lysis as described earlier [56]. DNA of engorged ticks was extracted using the Nucleospin Tissue kit (Macherey-Nagel, Düren, Germany) following the manufacturer's protocol for the purification of genomic DNA from insects. DNA of fleas from a previous study was extracted by disruption in liquid nitrogen with pestles followed by homogenization in 600 μl buffer RLT using a Qiashredder homogenizer according to the manufacturer's instructions (RNeasy minikit, Qiagen). DNA was extracted from 300 μl homogenate using the QIAamp DNA mini kit (Qiagen). DNA was eluted in 50 μl elution buffer. DNA of tissue samples from mice was extracted with the DNeasy Blood and Tissue kit (Qiagen, Hilden, Germany) according to the manufacturer's instructions.

### PCR-detection of Bartonella spp

Nymphal and adult vegetation ticks, larvae from Vrouwenpolder, ticks collected from cats and deer, deer keds, rodent tissue samples, fleas collected from rodents and pet animals were tested for the presence of *Bartonella *spp.. *Bartonella *spp. DNA was detected by PCR followed by sequencing as previously described (59). Briefly, the gltA gene was amplified using 5'-GGGGACCAGCTCAtGGTGG and 5'-AATGCAAAAAGAACAGTAAACA as primers, yielding amplification products of approximately 380 base pairs. A serial dilution of the cultivated *B. henselae *ATCC 49882 strain was used as positive control [57]. The highest dilution was used as a positive control in PCR and to spike tick- and flea lysates to identify samples that contain PCR-inhibitory components. Inhibition was negligible in all tick and flea samples. PCR amplification of parts of the 16S rRNA gene was done exactly as described by García-Esteban and colleagues, using 16S-R and P24Emod as primer pairs [58].

### PCR-detection of Rickettsia spp

**Figure 1:**
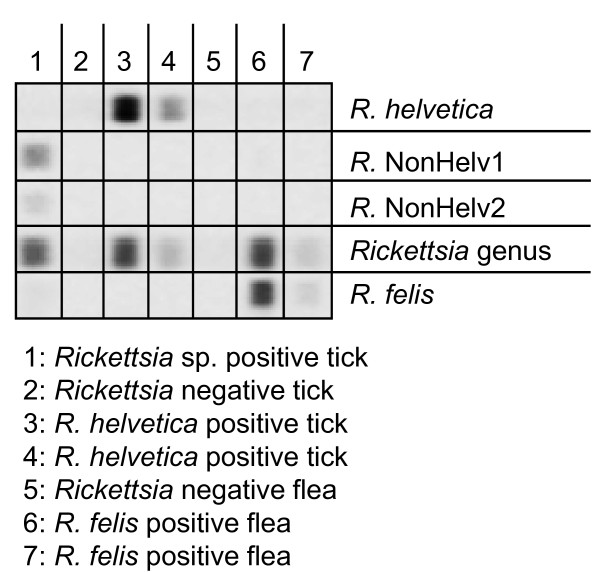
**Reverse Line Blot with *R. helvetica*, non-helvetica and *R. felis *probes**. PCR against a fragment of the 16S rRNA gene of *Rickettsia *was performed and samples were subjected to RLB as describe in the Methods section. Specificity of the probes is shown.

Tickets collected from cats as well as fleas from dogs and cats were tested for the presence of *Rickettsia *spp. DNA by PCR followed by reverse line blotting (RLB) as previously described [38] but with minor modification (Figure 1). Briefly, the 16S rRNA gene was amplified using 5'-AACGCTATCGGTATGCTTAACA and 5'-Biotin-ACTCACTCGGTATTGCTGGA as primers. For RLB analysis the following amino labeled probes were used: 5'-TTTAGAAATAAAAGCTAATACCG (catch all), 5'-CTTGCTCCAGTTAGTTAGT (*R. conorii*), 5'-GCTAATACCATATATTCTCTATG (*R. helvetica*), 5'-GTATATTCTCTACGGAAAAAAG (*Rickettsia sp. IRS3*), and 5'-TATATTCTCTACAGAGGAAAGATT (*R. felis)*. In order to detect potential double infections of ticks with *R. helvetica *and other rickettsial *species*, two RLB probes which were able to hybridize to DNA of most *Rickettsia *species except for *R. helvetica: *5'-AATACCGTATATTCTCTACGGA (NonHelv1) and 5'-AATACCGTATATTCTCTGCGGA (NonHelv2). Plasmids containing a 16S rRNA sequence from *R. helvetica, R.conorii, R. typhi *or *R. prowazekii *were used as positive controls.

Amplification of parts of the citrate synthase gene was done as described by De Sousa and colleagues using cs409d/rp1258n and cs535d/rp1258n as primer pairs [59].

### PCR detection of (Candidatus) Midichloria mitochondrii and phylogenetic analyses

Part of the 16S rRNA gene of "*Ca*. Midichloria mitochondrii" was amplified from tick lysates of larvae collected in Vrouwenpolder as described [60], using 5'-GCTACAGCTCTTGCCCGT (IrESF) and 5'-CAAAACCGACTCCCATGGC (IrESR) as primers. PCR amplicons were purified with the Qiaquick gel extraction kit (Qiagen Inc.) and sequenced using an ABI PRISM BigDye Terminator Cycle sequencing Ready Reaction kit (Perkin Elmer, Applied Biosystems). All sequences were confirmed by sequencing both strands. Sequences were compared with sequences in Genbank using BLAST. Phylogenetic analysis was performed using Bionumerics version 6.1 (Applied Maths, Gent, Belgium). Reference sequences were retrieved from Genbank. Sequences were aligned using Clustal X and distance-based analyses were conducted using Kimura 2-parameters distance estimates and trees were constructed using the Neighbour-Joining (NJ) algorithm, implemented in the MEGA program version 4.0. Bootstrap proportions were calculated by the analysis of 1000 replicates for NJ trees.

With the exception of the 16S rRNA PCR done for *Rickettsia *sp., which were analyzed by RLB, all PCR products were separated and analyzed by TAE agarose gel-electrophoresis. To minimize cross contamination and false-positive results, positive and negative controls were included in each batch tested for *Bartonella *or *Rickettsia *by the PCR. In addition, DNA extraction, PCR mix preparation, sample addition, and PCR analysis were performed in separated, dedicated labs.

## Results

### Vegetation ticks

To investigate the presence of *Bartonella henselae *in the Dutch tick population 1527 *Ixodes ricinus *nymphal and adult ticks from 16 geographically different locations were collected from recent field studies (Table 1).

Vegetation types of most sampling areas have been described previously with the exception of Vrouwenpolder. Vrouwenpolder is a vegetation rich dune area with several species of deciduous trees and shrubs and approximately 70% of the soil was covered with vegetation litter. Only very scarce and incomplete information is available about the fauna of all locations. Nymphs (98%) and adults (2%) were tested for *Bartonella *spp. by PCR on the gltA gene. PCR products of the expected size of approximately 380 bp were observed in 177 tick lysates. Sequencing was successful on 98 of these samples. Except for five sequences with one or two point mutations, all these sequences were identical, and 99-100% homologous (372 bp) to an uncultured *Bartonella-*related sp. isolate from *I. scapularis *(Genbank accession number EF662054). Neither *B. henselae *nor any other *Bartonella *species were detected in any of the lysates of these questing ticks.

**Figure 2:**
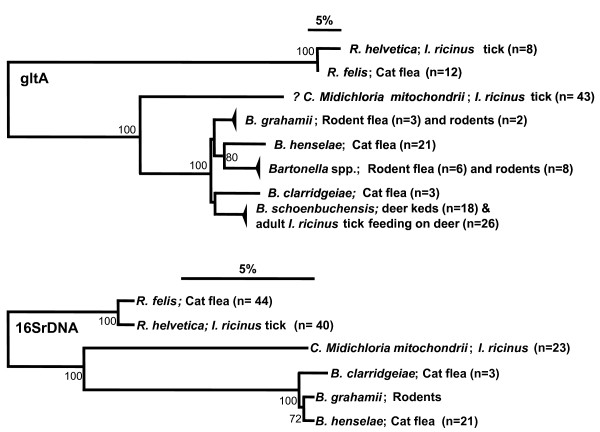
**Phylogenetic analysis of the microorganisms found in ectoparasites**. Neighbor-joining trees were based on the fragments of the gltA and 16S rRNA genes of the microorganisms found in the ectoparasites and rodents described in this study. Sequences were aligned and analysed as described in the Methods section. The number of sequenced isolates is between brackets. Small triangles indicate less than 1% variation. Only bootstrap values >75 are indicated. The *Bartonella*-related sp. found in ~ 10% of the questing ticks is indicated in the gltA-tree as *"? Ca. Midichloria mitochondrii*".

Next, lysates from 192 questing *I. ricinus *larvae from Vrouwenpolder were subjected to the same PCR against the gltA-gene. 41 larvae were positive and their sequences were identical to the *Bartonella-*related sp. from nymphs and adults. Under normal conditions, questing larvae have not yet had a blood meal. Transstadial but not transovarial (vertical) transmission of *Bartonella *has been described in ticks [33]. Therefore, it is unlikely that the *Bartonella-*related sp. in larvae is directly derived from a vertebrate host. The sequences of the *Bartonella-*related sp. might be derived from another microorganism present in ticks, which is capable of vertical transmission. This is supported by the fact that gltA-gene of this *Bartonella-*related sp. does not cluster with other *Bartonella *species (Figure 2). Therefore, the presence of the endosymbiont "*Ca*. Midichloria mitochondrii" in the same larval tick lysates was tested by PCR and sequencing using primers specifically against part of the 16S rRNA gene of "*Ca*. Midichloria mitochondrii" [61]. 56 tick lysates yield a PCR product of the expected size (1250 bp), which successful sequences (n = 43) were all identical to sequences designated to "*Ca*. Midichloria mitochondrii" (AJ566640). An additional, independent PCR using generic primers on the 16S rRNA of *Bartonella *species was performed [58] on the same 192 samples. Five of these samples yielded a PCR product of the expected size (440 bp). Sequencing of these products yielded 5 identical sequences (372 bp) which were 99% similar to a plethora of uncultured bacteria in Genbank, varying from an uncultured *Bartonella *isolated from the gut of *Apis mellifera mellifera *(EU055544), *Phyllobacterium myrsinacearum *(HQ380017) to a presumed *Bartonella grahamii *isolated from *Apodemus agrarius *(AB529498). As so many different microorganisms had similar homology, we concluded that this part of 16S rRNA gene is not suitable for detection and identification of *Bartonella *species.

### Ticks from cats

As domestic cats are the major reservoir host of *B. henselae*, adult ticks may acquire this pathogen while feeding on cats. 265 adult *I. ricinus *ticks removed from domestic cats were tested by PCR. *B. henselae *could not be detected in any of these tick lysates. In 56 lysates a fragment of approximately 380 bp was observed. Sequencing revealed that these samples contained the same *Bartonella-*related *sp*. as found in questing ticks (Table 2). The tick lysates from cats were also tested for *Rickettsia*e by PCR [62]. PCR products were analyzed by Reverse Line Blotting (RLB), which could differentiate several *Rickettsia *species, including *R. felis *(Figure 1). In 215 lysates *R. helvetica *was detected (Table 2). No other *Rickettsia *species were identified.

**Table 2:** *Bartonella *and *Rickettsia *in ticks feeding on cats

Microorganism (DNA)	Positive ticks (%)
*Bartonella henselae*	0 (0-1.4%)
*Bartonella-*related *sp*.	56 (16-27%)
*Rickettsia helvetica*	215 (76-86%)
*Rickettsia felis*	0 (0-1.4%)

**Number of ticks tested**	**265**

Adult *I. ricinus *ticks were removed from cats tested and tested for *Bartonella *and *Rickettsia *by PCR. *Rickettsia *species were identified by RLB. For *Bartonella*, the PCR products of 380 bp were sequenced and analysed. Six ticks were positive for both *R. helvetica *and *Bartonella-*related sp..

### Deer keds and ticks from deer

The absence of *Bartonella *in questing ticks seemed contradictory to previous findings in the Netherlands [63], where more than 70% of the ticks collected from red deer were positive for *Bartonella *or closely related species. Therefore, 96 lysates from adult ticks fed on red deer were analysed by PCR on the gltA gene. Indeed, the PCR products of 72 lysates (75%) yielded a 380 bp on the agarose gels. Sequencing revealed that at least 26 ticks contained DNA identical to *B. schoenbuchensis *(AJ564632), and 13 ticks contained the *Bartonella-*related sp. Sequencing was not successful in all cases, probably also because of mixed sequences of both *Bartonella-related *sp and *B. schoenbuchensis *(not shown). As *B. schoenbuchensis *was never detected in questing ticks (Table 1), we wondered how *B. schoenbuchensis *was transmitted between red deer. In the literature, deer keds (*Lipoptena cervi*) have been suggested to transmit *B. schoenbuchensis *between red deer as they have shown to be infected with *B. schoenbuchensis *[64]. We tested whether this was also the case in the Netherlands. For this, 56 deer keds were collected, lysed and a PCR on part of the gltA gene was performed. 46 deer keds were found to be positive of *B. schoenbuchensis *(AJ564632).

### Tissue samples from rodents

The origin of the *Bartonella-*related sp. from questing ticks was further investigated. One of the most prominent vertebrate hosts of *I. ricinus *are probably rodents. To determine whether mice are a potential reservoir of this *Bartonella-*related *sp.*, 96 DNA samples from ear tissue of small rodents, *Myodes glareolus *and *A. sylvaticus*, from the Duin en Kruidberg area were tested by PCR and sequencing a 380 bp part of the gltA gene. Twelve rodent samples were positive, but sequencing revealed that these sequences (n = 5) were 98-99% similar to *Bartonella *isolates from *A. flavicollis *(yellow-necked mouse) from Slovenia (347 bp, DQ155393) and Greece (338 bp, AY435110). The *Bartonella *sequences from these rodents were only 70% similar to the *Bartonella-*related sp. from questing ticks (Figure 2).

### Fleas from rodents

PCR of the gltA-gene on 24 flea lysates from wild rodents, *M. glareolus *and *A. sylvaticus*, caught in the Netherlands revealed that the *Bartonella sp*. found in *A. sylvaticus *was similar to those found in their fleas (Figure 2). Since the *Bartonella *sequences found in mouse were similar to those found in their fleas, but not similar to the *Bartonella *sequences found in questing *I. ricinus *ticks, these data suggested that the *Bartonella *found in rodents are most likely transmitted via fleas, not ticks.

### Fleas from pet animals

Although the DNA of a laboratory *B. henselae *strain was used as a positive control, we wondered whether the gltA PCR was specific and sensitive enough to detect *B. henselae *in questing ectoparasites. For this, fleas collected from pets were also tested under the same conditions as the tick lysates for both *Bartonella *and *Rickettsia*. 32 of 204 pools of cat fleas (*Ctenocephalides felis*) were positive for *B. henselae*. Three pools were positive for *B. clarridgeiae *and 43 pools for *R. felis *(Table 3). The identity of the latter two was confirmed by PCR and sequencing parts of the 16S rRNA and gltA genes, respectively (Figure 1). In one out of 17 pools of dog fleas (*Ctenocephalides canis*) DNA of *R. felis *was detected. No *Bartonella *sp. was detected in dog fleas. One third of the sampled dogs (n = 44) and cats (n = 109) carried fleas positive for *R. felis*, and one sixth of the animals had fleas positive for *B. henselae *(Table 4). Three cats had fleas positive for *B. clarridgeiae*.

**Table 3:** Pathogens found in fleas from pets

	*C. canis*	*C. felis*
Total (number)	48	528
Pools (size)	17 (1-7)	204 (1-17)
*B. henselae*-positive (pools)	0	32
*B. clarridgeiae*-positive (pools)	0	3
*R. felis*-positive (pools)	1	43

*Ctenocephalides canis and C. felis *fleas were separated based on morphological markers and were pooled per animal and flea species. In several cases more than one pool per animal was taken. Fleas were analysed by PCR and sequencing for the presence or absence of DNA from *Bartonella *and *Rickettsia *species.

**Table 4:** Prevalence of pets with fleas carrying zoonotic pathogens

	Dogs	Cats
Total	44	109
*R. felis*	13 (30%)	34 (31%)
*B. henselae*	7 (16%)	17 (16%)
*B. clarridgeiae*	0	3 (3%)
*R. felis *&*B. henselae*	2	3

Data are from Table 3, but now presented as prevalence in cats and dogs with fleas.

## Discussion

One of the major aims of this study was to investigate whether questing *I. ricinus *ticks transmit *Bartonella *species, particularly *B. henselae*, to humans [31,65,66]. In the 1719 questing ticks that were analysed, *B. henselae *was not found (Table 1). A gltA sequence, which was found in approximately 10% of the nymph and adult ticks, was closely related to a sequence found in *I. scapularis *ticks [67]. This sequence was designated previously as a *Bartonella *sp.. Phylogenetic analysis showed that the gltA sequence of this *Bartonella-*related species did not cluster with other *Bartonella *species (Figure 2). Furthermore, this *Bartonella-*related sequence was also found in lysates from 41 of 192 questing *I. ricinus *larvae, implying that the sequence is either from an environmental contamination or from a microorganism that is transmitted transovarially. To the best of our knowledge, transovarial transmission of *Bartonella *has never been demonstrated. Our data support this: *B. schoenbuchensis *was found in 75% of the adult ticks feeding on deer, but never in the questing larvae or nymphs tested in this study. We conclude that it is unlikely that the *Bartonella-*related sequence found in ticks is from a *Bartonella *species. Instead, we propose that the gltA sequence found in the questing *I. ricinus *ticks is from "*Ca*. Midichloria mitochondrii", an endosymbiont found in various hard ticks, including *I. ricinus *[61]. Only a few genes, but not the gltA gene, of this microorganism have been amplified and sequenced [68]. The presence of "*Ca*. Midichloria mitochondrii" in Dutch *I. ricinus *ticks was shown here by PCR and sequencing part of its 16S rRNA gene (Figure 2). "*Ca*. Midichloria mitochondrii" is present in mitochondria of tick cells and cannot be isolated or cultured. However, it cannot be excluded that the gltA gene isolated from tick lysates is unrelated to "*Ca*. Midichloria mitochondrii".

In this study, we have identified several *Bartonella *species: *B. henselae *and *B. clarridgeiae*, *B. grahamii *and another rodent-related *Bartonella *species and *B. schoenbuchensis*. As far as we know, the presence of these *Bartonella *species in the Netherlands is described here for the first time, except for *B. henselae *[69]. None of these were detected in this study in questing *I. ricinus *ticks. Statistically, less than 0.2% of the questing ticks might be infected with *B. henselae *or any other *Bartonella *species (95% exact binomial confidence interval). Therefore the risk of contracting bartonellosis from *I. ricinus *ticks in the Netherlands seems to be very low.

Questing *I. ricinus *larvae were subjected to PCR using generic primers on the 16S rRNA of *Bartonella *species [64]. Five of these samples were positive and yielded 5 identical sequences which were 99% similar to a plethora of uncultured bacteria in Genbank, varying from an uncultured *Bartonella *isolated from the gut of *Apis mellifera mellifera*, *Phyllobacterium myrsinacearum *to a presumed *Bartonella grahamii*. This high variety of hits indicates that the 16S rRNA gene has too little resolution power to positively identify a *Bartonella *species or to distinguish *Bartonella *from other, closely related genera. Furthermore, results of a BLAST depend on the quality of sequences in the database. In an earlier publication we discussed the pitfalls of using a database with only verified sequence types which neglects large amounts of less well verified but valuable data [70]. Using a large and freely accessible database like that of NCBI, however, has its own pitfalls as the quality of submitted sequences is not always good and identities of sequenced species are not always verified. *Bartonella *sp., *Rickettsia *sp. and "*Ca*. Midichloria mitochondrii" are very difficult to culture and some species have not yet been cultured at all. For uncultured microorganisms often only a limited number of genes are known and based on the limited data available it can be difficult to identify a microorganism to genus level. This can lead to misnaming of database entries which in turn can lead to further misidentifications [70].

While ticks seem to play a negligible role in *Bartonella *transmission, other ectoparasites, especially fleas, might be involved in the enzootic cycle of *Bartonella *in animals. *B. henselae *and *B. clarridgeiae *were found in cat fleas, and *B. grahamii *and another rodent-related *Bartonella *species were found in fleas collected from rodents, and *B. schoenbuchensis *in deer keds. All these ectoparasites could transmit *Bartonella *between animals and maybe also from animals to humans. *B. henselae *is well known to be associated to human cases of bartonellosis in the form of cat scratch disease [7,8]. It is mainly transmitted directly from cats to humans but might also be transmitted by infected cat fleas [19]. To date, including the current study, the transmission of *B. henselae *to humans via ticks has not been proven and the risk of transmission by tick is probably negligible [71]. *B. clarridgeiae *has also been frequently reported from cats and their ectoparasites [17,72] but has so far only incidentally been reported from human cases [20,22]. Cat fleas could play a role in *B. clarridgeiae *transmission but the route of transmission has not yet been established.

*B. schoenbuchensis *has been hypothesized to be involved in the development of deer ked dermatitis, a sometimes long-lasting skin condition following bites of deer keds [73]. While the exposure of the general population to deer keds is rather low, some specific groups like forest workers and hunters are at high risk to be bitten by these arthropods. In this limited population, deer keds might play a role in transmission of *B. schoenbuchensis*. In the general population exposure to *B. henselae *is much more likely than exposure to any of the other discussed *Bartonella *species. It is therefore also likely to cause most bartonellosis cases. Further research is necessary to determine the risk of zoonotic transmission of different *Bartonella *species upon a bite of cat fleas and other arthropods.

## Conclusions

Risk of human bartonellosis transmitted by *I. ricinus *ticks is negligible while other arthropods, including deer keds and fleas, can potentially transmit *Bartonella *sp. to humans. Earlier reports of high infection rates of questing ticks with a *Bartonella *species might in fact be due to the misidentification of "*Ca*. Midichloria mitochondrii" as *Bartonella *sp..

## Competing interests

The authors declare that they have no competing interests.

## Authors' contributions

FJ, WT, JRR, HS and PAMO were involved in study design. FG, EKEH, AMN and PO collected ticks and fleas, contributed to laboratory analyses and analyzed data. ETK and MF developed new methodology, performed laboratory analyses and analyzed data. HS acquired funding, was involved in data analyses and wrote the initial draft. ETK performed phylogenetic analyses and wrote the final draft. All authors were involved in completing the manuscript and approved the final version
